# Effects of Chitosan/Collagen Peptides/Cinnamon Bark Essential Oil Composite Coating on the Quality of Dry-Aged Beef

**DOI:** 10.3390/foods11223638

**Published:** 2022-11-14

**Authors:** Songshan Zhang, Xiaobin Sun, Yuanhua Lei, Baozhong Sun, Peng Xie, Xiaochang Liu

**Affiliations:** Institute of Animal Sciences, Chinese Academy of Agricultural Sciences, Beijing 100093, China

**Keywords:** meat ageing, dry-ageing bag, coating, chitosan, plant extracts, bioactive peptides, microbial community, volatile compounds

## Abstract

The aim of this study was to evaluate the effects of the chitosan/collagen peptides/cinnamon bark essential oil composite coating on dry-aged beef. Chitosan (2%, *w*/*v*), collagen peptides (1%, *w*/*v*), and cinnamon bark essential oil (1%, *v*/*v*) were homogenized to obtain the coating. Beef samples were divided into three groups (traditional dry-ageing, in-bag dry-ageing, and coating and then dry-ageing) and dry-aged for 42 days. Physiochemical, microbial, and sensorial parameters of samples were determined during the dry-ageing process. There were no significant differences (*p* > 0.05) in pH values, shear force values, cooking loss, color, juiciness, tenderness, and flavor across groups. The total volatile base nitrogen value of the coating group was lower than those of the other two groups. Compared to traditional dry-ageing, in-bag and coating dry-ageing reduced (*p* < 0.05) many volatile compounds such as alcohols, aldehydes, ketones, and acetate. In-bag and coating dry-ageing had no impact on the fungal community, but changed the bacterial community by inhibiting *Pseudomonas*. This study demonstrates that the chitosan/collagen peptides/cinnamon bark essential oil coating reduces microbial spoilage during dry-ageing, and has a small influence on product quality.

## 1. Introduction

Dry-ageing is a known way to enhance the eating quality of meat, especially tenderness and flavor. It is a process where unpackaged carcasses/sub-primal cuts are aged under strictly controlled conditions of refrigerated temperature, relative humidity, and air velocity for a period of time [1]. During the process, water evaporation, proteolysis, and lipolysis occur to allow changes in flavor-related compounds (e.g., free amino acids, peptides, fatty acids, and sugars), resulting in a characteristic dry-aged flavor [1,2]. The production of dry-aged beef is costly, due to weight loss, trimmings of dehydrated surface, and microbial contamination. To overcome these problems, a high moisture-permeable package, which is made of polyamide mix, was used to produce dry-aged beef. In-bag dry-ageing reduces losses and microbial load, resulting in equivalent palatability attributes compared to traditional dry-ageing [3,4].

Recently, as the interest for green packages has increased, exploring natural package materials to improve the dry-ageing process is needed. Chitosan film is an alternative to the dry-ageing bag, due to its biodegradability, high water-vapor permeability, and antimicrobial property. Chitosan wrapping has been proved to reduce microbial counts and total loss compared to traditional dry-ageing [5]. Recently, studies have focused on incorporating plant essential oils [6,7,8] and peptides [9,10,11] into chitosan to improve the antimicrobial property of the chitosan film. These composite films showed better antimicrobial and antioxidant activities than pure chitosan film. 

Cinnamon is a commonly used spice in meat products. Cinnamon bark essential oil, mainly containing cinnamaldehyde, exhibited antibacterial activity in meat products [12,13,14]. Collagen is the main structural protein of by-products (e.g., connective tissues, bones, skins, and scales). Collagen peptides, the products of collagen hydrolysis, can be used as antimicrobial and antioxidant ingredients in food [15,16]. The aim of this study was to evaluate the effectiveness of a chitosan coating incorporated with cinnamon bark essential oil and collagen peptides in optimizing the dry-ageing process of beef, compared to the traditional dry-ageing and in-bag dry-ageing. pH value, shear force value, cooking loss, total volatile base nitrogen value, color, volatile compounds, microbial community, yield, and sensory evaluation were determined during the dry-ageing period of 42 days.

## 2. Materials and Methods

### 2.1. Preparation of Coating Solution

Chitosan solution (2%, *w*/*v*) was prepared by dissolving chitosan (deacetylation degree > 90%; Macklin, Shanghai, China) in 2% (*v*/*v*) acetic acid. Collagen peptides from bovine skin (<10 kDa; Gelita, Eberbach, Germany) were dissolved in distilled water to obtain a concentration of 2% (*w*/*v*). Chitosan and collagen peptides solutions were mixed in equal volumes and glycerol (10% of chitosan, *w*/*w*) was added to the mixture as the plasticizer. The mixture was stirred for 1 h to obtain a uniform solution. Then, Tween 80 (emulsifier) and cinnamon bark essential oil (1%, *v*/*v*; Xinsen, Ji’an, China) were incorporated, and the mixture was homogenized at 18,000 rpm for 2 min. 

### 2.2. Sample Preparation and Treatments

Striploins (left and right sides) from six Xinjiang Brown steer (24 months old) were purchased from a commercial beef plant (Chuangjin, Xinjing, China) at 72 h post-mortem. Each striploin was cut into six equal sections. All sections were randomly divided into three groups: coating group, in-bag group, and traditional group. For the coating group, samples were immersed in the coating solution for 30 s. The immersion operation was repeated three times at an interval of 10 s. For the in-bag group, samples were packed in commercial dry-ageing bags (UMAi Dry^®®^, Minneapolis, MN, USA) and vacuum-sealed. For the traditional group, samples were not treated. All samples were placed on the shelves in a dry-ageing room (2.5 ± 0.5 °C, relative humidity of 80 ± 5%, 0.5 m/s air velocity). Samples were obtained on days 0, 7, 14, 28, and 42. The dehydrated surfaces were trimmed and used for microbial community analysis. The remaining parts were used for analyses of other parameters. Samples used for volatile compounds and microbial community were stored at −80 °C until analysis.

### 2.3. pH Value

pH values of beef samples were determined using a pH meter (Hanna, Woonsocket, RI, USA) equipped with a penetration probe. The pH meter was calibrated using two standard buffers (pH 4.01 and 7.01).

### 2.4. Cooking Loss and Shear Force Value

Beef samples (5 cm × 4 cm × 3 cm) were placed in plastic bags and cooked in a water bath (80 °C) until the core temperature reached 72 °C. Weight before cooking and weight after cooking were recoded. Cooking loss was calculated as the following:

Cooking loss (%) = (weight before cooking − weight after cooking)/weight before cooking × 100%(1)

After being weighed, cooked beef samples were sent for shear force analysis. Six subsamples (the cross-section of 1 cm × 1 cm, 3 cm parallel to the fiber axis) were taken. The shear force value was determined perpendicular to the fiber orientation using a TA-XT Plus texture analyzer equipped with an HDP/BSW probe. The pre-test speed was 1.0 mm/s, the test speed was 1.0 mm/s, and the post-test speed was 10.0 mm/s. The trigger force was 5 g. The time interval was 5 s. For each sample, shear force values of six subsamples were averaged.

### 2.5. Total Volatile Base Nitrogen (TVB-N) Value

Ground beef samples (10 g) were homogenized with 100 mL of distilled water. After being shaken for 30 min, the mixture was centrifuged to obtain the supernatant. The supernatant was used for TVB-N determination using the semi-microtitration method. The TVB-N value was expressed as mg N/100 g flesh.

### 2.6. Color

The surface color of the sample was determined after blooming for 40 min at room temperature. For each sample, the CIE L* value, a* value, and b* value of three different locations on the surface were determined using a CR-400 colorimeter (Konica Minolta, Osaka, Japan). The colorimeter was calibrated using a white tile (Y = 93.4, x = 0.3157, y = 0.3322).

### 2.7. Volatile Compounds

Volatile compounds were determined by a FlavorSpec^®®^ gas chromatograph-ion mobility spectrometer (GC-IMS) (G.A.S. Instrument, Germany) equipped with an MXT-5 column (15 m × 0.53 mm × 1 μm) (Restek; Bellefonte, PA, USA). Beef samples were cooked on the pan until the core temperature reached 70 °C. The cooked samples were frozen with liquid nitrogen, and grinded. Two grams of the sample was weighed, sealed in a glass vial (20 mL), and incubated at 60 °C for 20 min. Then, 500 μL of gas was injected with a syringe at 85 °C. The column temperature was 60 °C and the IMS temperature was 45 °C. The carrier gas and drift gas were high-purity nitrogen. The flow rate of carrier gas was set as the following gradient: 0 min, 2 mL/min; 2 min, 2 mL/min; 10 min, 10 mL/min; 20 min, 100 mL/min; 30 min, 150 mL/min. The flow rate of carrier gas was 150 mL/min. Volatile compounds were identified by GC-IMS library search software.

### 2.8. Bacterial Community and Fungal Community

Microbial communities of samples (day 0 and 42) were analyzed. The total DNA was extracted using the FastDNA^®®^ Spin Kit for Soil (MP Biomedicals, Solon, OH, USA). The purity and concentration of DNA were examined using a NanoDrop 2000 (Thermo Fisher Scientific, Waltham, MA, USA). The integrity of DNA was tested on 1% agarose gel. The V3–V4 region of the 16S rDNA was amplified using primer 338F/806R. The PCR reaction mixture (20 μL) contained 10 ng of DNA template, 4 μL of 5 × FastPfu Buffer, 2 μL of dNTPs (2.5 mM), 0.4 μL of FastPfu Polymerase, 0.2 μL of BSA, 0.8 μL of each primer (5 μM), and ddH_2_O. The ITS1 region of fungal genes was amplified using primer ITS1F/ITS2R. The PCR reaction mixture (20 μL) contained 10 ng of DNA template, 2 μL of 10× Buffer, 2 μL of dNTPs (2.5 mM), 0.2 μL of rTaq Polymerase, 0.2 μL of BSA, 0.8 μL of each primer (5 μM), and ddH_2_O. The amplification condition was 95 °C for 3 min; 35 cycles of 95 °C for 30 s, 55 °C for 30 s and 72 °C for 45 s; 72 °C for 10 min. PCR products were sent to Majorbio Bio-Pharm Technology Co., Ltd. (Shanghai, China) for sequencing using an Illumina MiSeq PE300 platform.

### 2.9. Yield

On day 42, weight after ageing and weight after trimming were recorded. Ageing loss, trimming loss, and total loss were calculated as follows:

ageing loss (%) = (weight before ageing − weight after ageing)/weight before ageing × 100%(2)

trimming loss (%) = (weight before trimming − weight after trimming)/weight before trimming × 100%(3)

total loss (%) = (weight before ageing − weight after trimming)/weight before ageing × 100%(4)

### 2.10. Sensory Evaluation

Sensory evaluation was conducted by ten experienced assessors (six females and four males). Assessors were also screened and trained according to AMSA (2016) [17] guidelines, and had more than two years’ experience in sensory evaluation of meat. In addition, dry-aged beef of different ageing times and quality grades were served to assessors before this study so they could familiarize themselves with the attributes of dry-aged beef. Samples were heated on a preheated pan at 200 °C until the core temperature reached 70 °C. Heated samples were cut into cubes (2 cm × 2 cm × 1.5 cm) and kept in an incubator (70 °C) prior to sensory analysis. Assessors rated tenderness, juiciness, and flavor using an 8-point scale (1 = extremely tough, dry, and bland aged-beef flavor; 8 = extremely tender, juicy, and intense aged-beef flavor).

### 2.11. Data Analysis

Differences among means were assessed by analysis of variance and Duncan’s multiple range test at the level of 5% using SPSS 17.0 (SPSS Inc., Chicago, IL, USA). Data of microbial communities were analyzed on the online platform of Majorbio Cloud Platform (https://cloud.majorbio.com, accessed on 14 April 2022).

## 3. Results and Discussion

### 3.1. pH Value, Shear Force Value, and Cooking Loss

The initial pH value of beef was 5.49 ± 0.03 (Table 1). Differences in pH values were not evident among three groups during ageing, indicating that pH values were not influenced by coating and in-bag treatments.

The shear force value before ageing was 44.0 ± 4.3 N (Table 1). Shear force values of all groups declined remarkably (*p* < 0.05) on day 14, and afterward, no significant decrease was observed for all groups. Previous studies also reported that the effect of ageing on tenderness occurred mainly during the first three weeks [18,19]. It may be attributed to the decreased activity of the calpain system, which is primarily responsible for postmortem tenderization [20]. No significant differences in shear force values were found among groups. It demonstrated that in-bag and coating treatments did not influence beef tenderization during dry-ageing. 

Cooking losses of all groups decreased by 7.9–8.4% after 42 days of dry-ageing (Table 1). Likewise, Gudjónsdóttir et al. [5] reported that traditional and chitosan wrapping dry-ageing for 21 days decreased cooking losses by 9.0% and 10.8%. Macharáčková et al. [21] also found lower cooking losses of beef dry-aged for 21 days than those dry-aged for 5 days. Cooking loss of meat is caused by water expelling when muscle fibers and myofibrils shrink. The reason for the decreased cooking loss during ageing may be that the degradation of cytoskeletal proteins (vinculin, desmin, and talin) destructs linkages related to the shrinkage of muscle fibers and myofibrils, weakening the force expelling intracellular water [22]. Cooking losses of three groups were similar throughout the ageing period. Ahnström et al. [3] similarly reported no differences in cooking loss between in-bag dry-ageing and traditional dry-ageing.

### 3.2. TVB-N Value

TVB-N values increased during the ageing process (Table 1), in accordance with the result of Kang et al. [23]. No significant difference in TVB-N value was observed among treatments, except the value of day 42. On day 42, the TVB-N value of the coating group (16.69 ± 0.83 mg/100 g) was lower than those of the traditional group (18.62 ± 0.55 mg/100 g) and the in-bag group (18.01 ± 1.01 mg/100 g). As TVB-N is mainly produced by microorganisms, the lower TVB-N value in the coating group can be due to the control of microbial spoilage. Ojagh et al. [24] reported that the chitosan coating enriched with cinnamon oil delayed TVB-N production in rainbow trout by inhibiting bacterial growth or decreasing bacterial deamination of nonprotein nitrogen compounds.

### 3.3. Color

**Figure 1:**
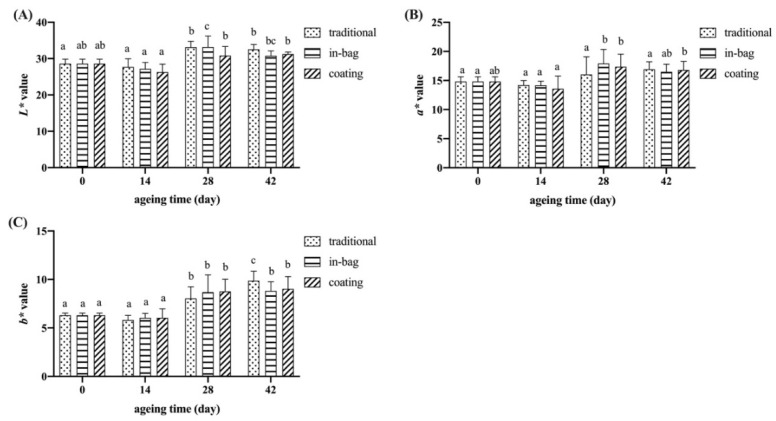
Effects of different treatments on L* (**A**), a* (**B**), and b* (**C**) values of dry-aged beef. ^a–c^ Means having same lowercase letters indicate no significant differences between ageing times for the same group, *p* > 0.05.

The CIE L*a*b* system was used to evaluate beef color through lightness (L*), redness (a*), and yellowness (b*). For all three groups, L* and a* values did not change significantly after 42 days of dry-ageing, while b* values increased slightly (Figure 1). Hulánkováa et al. [25] detected no changes in L*, a*, and b* values after 13–36 days of dry ageing. Vossen et al. [19] found that a* and b* values increased after six weeks of dry ageing, and decreased after nine weeks of dry ageing. Meat color changes during ageing due to many factors, such as myoglobin concentration and stability [26], light reflection related to the amount of free water [27], and proteins conformation [28]. No differences in L*, a*, and b* values were detected between ageing treatments, indicating that the in-bag and coating had no adverse effects on meat color.

### 3.4. Volatile Compounds

**Figure 2:**
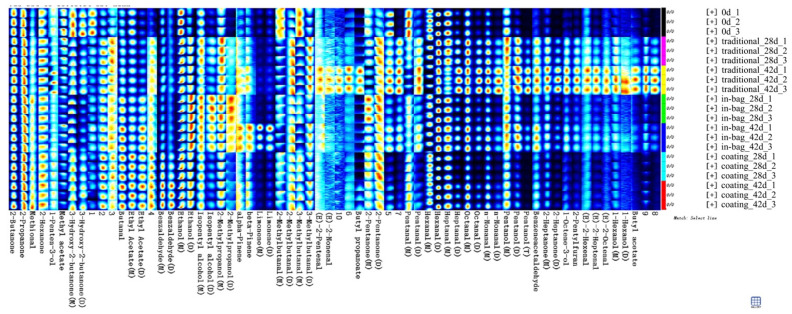
Effects of different treatments on volatile compounds of dry-aged beef.

Volatile compounds of beef change during the dry-ageing process and subsequent cooking. As shown in Figure 2, after 42 days of traditional dry-ageing, dramatic increasing trends were detected in aldehydes (3-methylbutanal, 2-methylbutanal, benzene acetaldehyde, E-2-heptenal, nonanal, octanal, heptanal, and pentanal), ketones (2-heptanone), alcohols (1-octene-3-ol, pentanol, 2-methylpropanol, and ethanol), furans (2-pentylfuran), and esters (butyl acetate and butyl propanoate). Similarly, Lee et al. [29] and Li et al. [30] reported that dry-ageing increased concentrations of aldehydes, ketones, and alcohols. 3-methylbutanal (malty odor), 2-methylbutanal (chocolate odor), and benzene acetaldehyde (rosy odor) come from the Strecker degradation of leucine, isoleucine, and phenylalanine, respectively [31,32]. The increase in Strecker aldehydes may be attributed to the release of their respective precursors during proteolysis. Straight aldehydes of C_5_–C_9_ carbons (green, oily, and fatty odor), 2-heptanone (fruity, floral, and cheese odor), 1-octene-3-ol (mushroom odor), and 2-pentylfuran (green bean and butter) are mostly oxidized from fatty acid [31,32,33]. Esters from C_1_–C10 contribute to the fruity flavor and are produced through the esterification of alcohols and carboxylic acids by microorganisms [31]. 

Compared to the traditional group, the in-bag group had lower concentrations of nonanal, octanal, heptanal, pentanal, 2-heptanone, 1-octene-3-ol, 2-pentylfuran, butyl propanoate, and butyl acetate, and higher concentrations of limonene, β-pinene, isopentyl alcohol, 2-methylpropanol, ethanol, ethyl acetate, and butanal. Barragán-Hernández et al. [34] also reported that in-bag dry-ageing impacted only a few volatile compounds. Setyabrata et al. [35] studied the volatile profiles of traditional dry-aged beef and in-bag dry-aged beef, and detected differences in n-aldehyde, ketones, esters, alcohols, and heterocyclic compounds. The reason may be that in-bag dry-ageing limited the moisture evaporation and oxygen transmission, thus affecting the concentration and oxidation of flavor precursors. 

Compared to the traditional group, some compounds accumulated less in the coating group, including 1-octene-3-ol, 2-pentylfuran, 2-heptanone, benzene acetaldehyde, pentanol, nonanal, octanal, heptanal, pentanal, methional, butyl propanoate, and butyl acetate. Benzaldehyde, ethyl acetate, and butanal in the coating group were higher than the traditional group. Most components of cinnamon bark oil were not detected in the coating group. The reason may lie in three aspects: (ⅰ) the coating solution existed in the outer part of beef and did not permeate into the inner part during ageing; (ⅱ) the dehydrated outer part was trimmed; (ⅲ) components of cinnamon bark oil degraded at the high temperature of pan-frying. For example, cinnamaldehyde, the main component of cinnamon bark oil, could degrade to benzaldehyde above 60 °C [36].

### 3.5. Microbial Community

**Figure 3:**
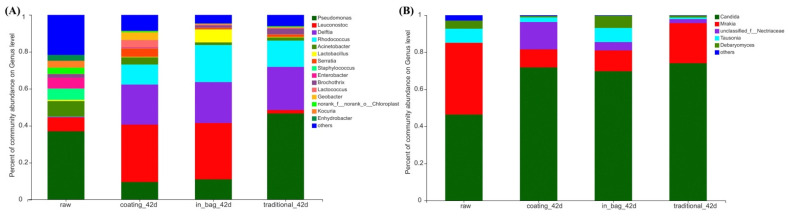
Effects of different treatments on bacterial community (**A**) and fungal community (**B**) of dry-aged beef at genus level.

The V3–V4 region of bacterial 16S rDNA was sequenced. A total of 107,166 sequences (average length 424 bp) were obtained from fresh beef, 170,324 sequences (average length 425 bp) from the coating group, 162,779 sequences (average length 424 bp) from the in-bag group, and 168556 sequences (average length 425 bp) from the traditional group. As shown in Figure 3A, the initial bacterial community was dominant by *Pseudomonas* (37.05%), *Acinetobacter* (8.40%), *Leuconostoc* (7.45%), *Staphylococcus* (6.13%), and *Enterobacter* (5.83%). After 42 days of dry-ageing, the dominant genera in the traditional group were *Pseudomonas* (46.68%), *Delftia* (23.31%), and *Rhodococcus* (14.20%). The dominant genera in the in-bag group were *Leuconostoc* (30.46%), *Delftia* (22.15%), *Rhodococcus* (20.03%), *Pseudomonas* (11.05%), and *Lactobacillus* (7.05%). Similarly, the dominant genera in the coating group contained *Leuconostoc* (31.05%), *Delftia* (21.70%), *Rhodococcus* (10.81%), and *Pseudomonas* (9.59%). The proportions of *Pseudomonas* were lower (*p* < 0.05) in the in-bag group and coating group than the traditional group. This indicated that in-bag dry-ageing and coating dry-ageing inhibited the growth of *Pseudomonas*. *Pseudomonas* is commonly detected in dry-aged beef [37,38,39]. *Pseudomonas* could partly out-compete lactic acid bacteria [40]. The lower proportion of *Pseudomonas* may lead to the higher proportion of *Leuconostoc* in the in-bag group and coating group. 

The relative abundance of *Pseudomonas* (r^2^ = 0.8786, *p* = 0.0018) and *Brochothrix* (r^2^ = 0.7731, *p* = 0.0145) were significantly correlated with TVB-N value. Similar to *Pseudomonas*, the proportion of *Brochothrix* was lower (*p* < 0.05) in the coating group (0.08%) than the traditional group (2.99%). Many *Pseudomonas* species and *Brochothrix* species are associated with food spoilage, such as *P. fragi*, *P. psychrophila,* and *B. thermosphacta*. These species could produce large amounts of TVB-N [41,42,43]. The lower TVB-N value of the coating group on day 42 may be explained by the inhibition effect of coating treatment on *Pseudomonas* and *Brochothrix*. Chitosan [44,45] and cinnamon essential oil [46,47] were effective in preventing the growth of *Pseudomonas* and *Brochothrix* in meat.

The ITS1 region of fungi was sequenced. A total of 143,518 sequences (average length 208 bp) were obtained from fresh beef, 219,573 sequences (average length 184 bp) from the coating group, 226,556 sequences (average length 190 bp) from the in-bag group, and 200,034 sequences (average length 187 bp) from the traditional group. As shown in Figure 3B, the initial fungal community was dominant by *Candida* (46.41%), *Mrakia* (38.70%), and *Tausonia* (7.59%). After 42 days dry-ageing, the dominant genera in the traditional group were *Candida* (74.11%) and *Mrakia* (21.65%). The dominant genera in the in-bag group were *Candida* (69.78%), *Mrakia* (11.23%), *Tausonia* (7.61%), and *Debaryomyces* (6.63%). The dominant genera in the coating group were *Candida* (71.84%), *Mrakia* (9.74%), and unclassified_f_Nectriaceae (14.76%). In-bag dry-ageing and coating dry-ageing showed little effect on the fungal community. *Candida* and *Debaryomyces* have been commonly detected in dry-aged beef [37,48]. A *Debaryomyces* species, *Debaryomyces hansenii*, isolated from dry-aged beef could produce free amino acids and fatty acids through proteolysis and lipolysis [49].

### 3.6. Yield (Ageing Loss, Trimming Loss, and Total Loss)

**Figure 4:**
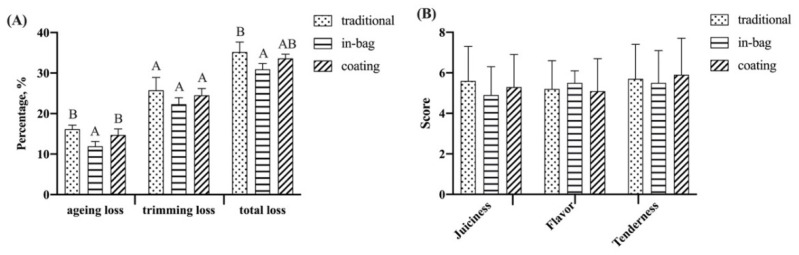
Effects of different treatments on yield (**A**) and sensory score (**B**) of beef dry-aged for 42 days. ^A,B^ Means having same capital letters indicate no significant differences between groups, *p* > 0.05.

Dry-ageing resulted in losses due to water evaporation (ageing loss) and trimming of the dried surface (trimming loss). The ageing loss, trimming loss, and total loss of beef after 42 days of dry-ageing are shown in Figure 4A. Ageing loss was higher in the traditional group (16.15 ± 1.00%) and coating group (14.71 ± 1.52%) than the in-bag group (11.92 ± 1.18%). Trimming loss (22.33–25.74%) showed no significant differences among three groups. Total losses of the traditional group (35.17 ± 2.47%) and coating group (33.61 ± 1.05%) were higher than the in-bag group (30.19 ± 1.45%). Compared to traditional dry-ageing, in-bag ageing decreased ageing loss by 3.23% and total loss by 4.98%. Shi et al. [50] found that in-bag dry-ageing controlled the level of weight loss and total loss during dry-ageing, depending on the moisture permeability of bags. The inhibition effects of the coating on yield were not significant compared to traditional dry-ageing. A contrary result was reported by Gudjónsdóttir et al. [5], who found that electrospun chitosan fiber wrapping reduced ageing loss, trimming loss, and total loss compared to traditional dry-ageing. The reason for different results may be that the chitosan wrapping material prepared by electrospinning has a smaller pore size, and, thus, is more resistant to water evaporation than the chitosan film produced by the coating.

### 3.7. Sensory Evaluation

Scores of juiciness, tenderness, and flavor showed no significant difference across all groups (Figure 4B). Ahnström et al. [3] and DeGeer et al. [51] also reported no significant differences in sensory attributes between traditional dry-aged beef and in-bag dry-aged beef. Cinnamon bark essential oil exhibited an intense odor. However, the coating group showed only a slight flavor of cinnamon bark and its effect on dry-aged beef flavor was little. The reason may be that the outer parts of beef, which directly contacted the coating solutions, were trimmed. In addition, the cooperation of cinnamon bark essential oil with chitosan could control the release of essential oil, avoiding the negative organoleptic effects caused by the direct addition of essential oil [52].

## 4. Conclusions

Compared to traditional dry-ageing and in-bag dry-ageing, the chitosan/collagen peptides/cinnamon bark essential oil composite coating inhibited *Pseudomonas* growth and TVB-N production, without affecting the fungal community, pH value, cooking loss, color, and shear force. The composite coating had no significant effects on flavor, although it changed the volatile profile of dry-aged beef. These results suggest that the chitosan/collagen peptides/cinnamon bark essential oil coating can be developed as a low-cost, natural, and anti-microbial package when producing dry-aged beef. Future work should focus on regulating the release of functional compounds to maximize the efficiency of the coating. Additionally, the efficiency of the coating in other meat products can be explored.

## Figures and Tables

**Table 1:** Effects of different treatments on pH value, cooking loss, shear force value, and TVB-N value of beef during dry-ageing.

	Treatment	Ageing Time (Day)			
		0	14	28	42
pH value	Traditional	5.49 ± 0.03 ^a^	5.50 ± 0.02 ^a^	5.60 ± 0.02 ^b^	5.56 ± 0.04 ^b^
	In-bag	5.49 ± 0.03 ^a^	5.55 ± 0.04 ^ab^	5.61 ± 0.06 ^b^	5.53 ± 0.06 ^ab^
	Coating	5.49 ± 0.03 ^a^	5.59 ± 0.09 ^a^	5.60 ± 0.07 ^a^	5.57 ± 0.07 ^a^
Cooking loss, %	Traditional	18.8 ± 1.6 ^b^	15.9 ± 4.7 ^b^	11.1 ± 2.4 ^a^	10.4 ± 1.1 ^a^
	In-bag	18.8 ± 1.6 ^c^	13.9 ± 2.5 ^b^	10.5 ± 1.7 ^a^	10.5 ± 1.2 ^a^
	Coating	18.8 ± 1.6 ^b^	14.1 ± 3.5 ^a^	12.1 ± 3.3 ^a^	10.9 ± 3.4 ^a^
Shear force value, N	Traditional	44.0 ± 4.3 ^b^	24.6 ± 3.6 ^a^	27.8 ± 5.7 ^a^	29.8 ± 2.9 ^a^
	In-bag	44.0 ± 4.3 ^c^	29.1 ± 4.9 ^ab^	22.9 ± 5.3 ^a^	33.4 ± 5.2 ^b^
	Coating	44.0 ± 4.3 ^b^	28.5 ± 7.9 ^a^	29.8 ± 8.3 ^a^	28.4 ± 7.0 ^a^
TVB-N value, mg/100 g	Traditional	10.06 ± 0.58 ^a^	12.86 ± 0.94 ^b^	18.60 ± 1.11 ^c^	18.62 ± 0.55 ^cB^
	In-bag	10.06 ± 0.58 ^a^	12.92 ± 1.18 ^b^	19.06 ± 3.19 ^c^	18.01 ± 1.01 ^cB^
	Coating	10.06 ± 0.58 ^a^	13.18 ± 0.53 ^b^	18.08 ± 0.21 ^d^	16.69 ± 0.83 ^cA^

Mean ± standard deviation; ^a–d^ Means having the same superscript lowercase letters within a row indicate no significant differences, *p* > 0.05; ^A,B^ Means having the same superscript capital letters within a column indicate no significant differences, *p* > 0.05.

## Data Availability

Data is contained within the article.
